# A Lymphocytic Variant of Hypereosinophilic Syndrome Presenting With Isolated Cutaneous Manifestations

**DOI:** 10.7759/cureus.29745

**Published:** 2022-09-29

**Authors:** Oumayma Kamal Idrissi, Fouzia Hali, FatimZahra El Fatoiki, Soumiya Chiheb

**Affiliations:** 1 Department of Dermatology and Venereology, Ibn Rochd University Hospital, Casablanca, MAR

**Keywords:** hypereosinophilic syndrome, erythroderma, palmoplantar pustulosis, prurit, eosinophilia, hyper

## Abstract

Hypereosinophilic syndrome (HES) is a rare disease defined by a persistent increase in eosinophilic cells associated with organ damage without any underlying cause. Three variants have been identified: myeloproliferative, lymphocytic, and idiopathic syndrome. The symptomatology is variable because it depends on the involvement of different organs, including the circulatory system, skin, lungs, digestive tract, peripheral and central nervous system, and eyes. Although cutaneous involvement may frequently reveal an underlying HES. We report a case of a 52-year-old man with a 12-year history of skin lesions with intense pruritus. On examination, the patient presented with erythroderma, extensive infiltrated plaques, excoriated itchy papules, palmoplantar pustules, ear infiltration, periorbital edema, and cutaneous xerosis.

Histopathology showed lichenoid dermatitis without epidermotropism. Inflammatory infiltrates in the dermis were principally composed of eosinophilic cells and lymphocytes. Serum immunoglobulin E and peripheral blood immunophenotyping showed atypical T lymphocyte proliferation CD4+CD3-,^ ^and clonal *TCR* gene rearrangement was in favor of lymphocytic HES. The patient was treated with prednisone (1 mg/kg/day) and pegylated interferon alpha with improvement.

This case shows that HES should be suspected in patients with dermatological lesions and hypereosinophilia, without obvious cause. Elimination of secondary causes of eosinophilia, evaluation of deep organ involvement, and cytogenetic studies to assess prognosis are paramount. Pegylated interferon alpha 2a may be an effective treatment option for steroid-resistant or refractory patients with lymphocytic HES.

## Introduction

Hypereosinophilic syndrome (HES) is an uncommon, multisystem, heterogeneous group of disorders with significant morbidity and mortality. It is characterized by persistently elevated peripheral blood eosinophilia associated with symptomatic organ involvement, excluding secondary causes of eosinophilia [1].

HES is classified as myeloproliferative, lymphocytic, and idiopathic [2]. The lymphocytic variant of HES (L-HES) is a distinct subtype, characterized by aberrant clonal T-cell populations that produce eosinophil-promoting cytokines [3].

We present a case of a lymphocytic variant of HES with cutaneous manifestations, treated with pegylated interferon alpha 2a.

## Case presentation

**Figure 1:**
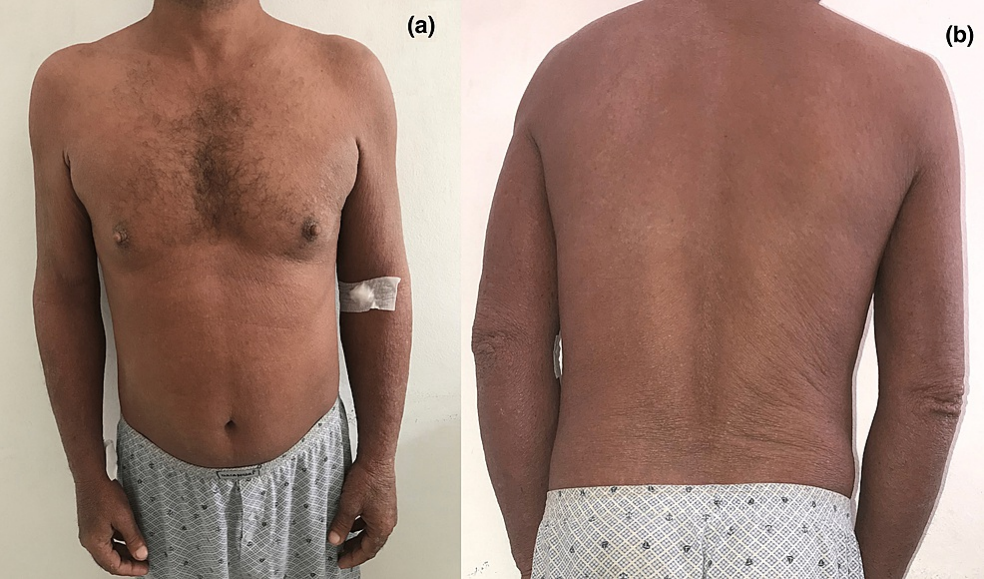
Erythroderma: (a) trunk and (b) back

**Figure 2:**
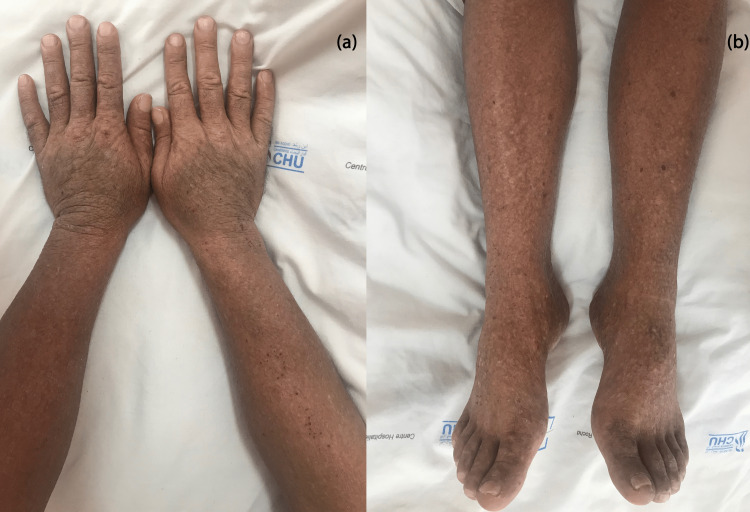
Infiltrated excoriated papules on (a) hands and (b) feet

**Figure 3:**
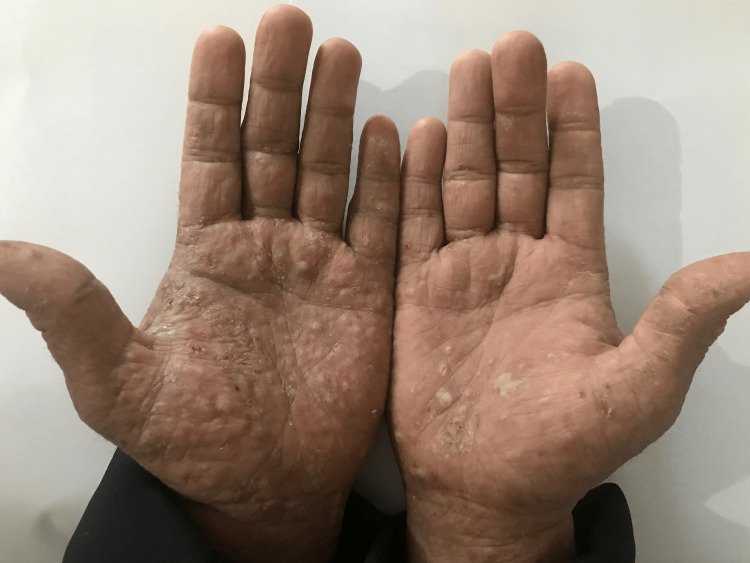
Papules and pustules on the palmar surface

A 52-year-old male presented with a three-year history of periodic pruritic and generalized erythema that had been diagnosed as mycosis fungoid, which was suspected in a single histological study and whose immunohistochemical study did not allow the diagnosis to be fully retained. He had received many treatments, such as systemic and topical corticosteroids, systemic methotrexate, and antihistamines, with no improvement. Physical examination showed erythroderma with extensively infiltrated plaques (Figure 1), excoriated itchy papules (Figure 2), palmoplantar pustules (Figure 3), ear infiltration, periorbital edema, and cutaneous xerosis with multiple axillary lymphadenopathies. There was no abdominal organomegaly and examination for central nervous, respiratory, cardiovascular, and musculoskeletal systems was normal.

The patient’s eosinophil count increased to 3.1 × 109/L. No Sézary abnormal cells were observed in peripheral blood in two separate samples.

Hepatitis serology, human T-lymphotropic virus type I (HTLV-I), and HIV antibodies were negative. No antinuclear antibody or antineutrophil cytoplasmic antibody was detected. Stool examination for parasites was negative. Serum immunoglobulin E was elevated at 3382. Peripheral blood immunophenotyping showed atypical T lymphocyte proliferation CD4+CD3-, and T-cell receptor rearrangement studies revealed a clonal population of T lymphocytes.

Computed tomography of the chest and ultrasonography of the abdomen showed normal findings. Echocardiography and electrocardiogram were normal. Skin biopsy showed nonspecific lichenoid dermatitis without epidermotropism. Inflammatory infiltrates consisted mainly of lymphocytes and eosinophils. Myelogram and bone marrow biopsy showed moderate eosinophilia without evidence of lymphoma. A lymph node biopsy demonstrated dermopathic lymphadenitis but no evidence of malignancy.

The diagnosis of lymphocytic HES was retained. The patient was treated with prednisone 1 mg/kg/day without significant improvement. Pegylated interferon alpha 2a administered subcutaneously weekly was added. Two months later, we noted an important resolution of cutaneous lesions and a decrease in eosinophilia.

## Discussion

HES was introduced by Hardy and Anderson in 1968, and Chusid et al. suggested diagnostic criteria for HES in 1975. These criteria comprised chronic, longer than six months hypereosinophilia (HE) (>1.5 × 109/L) with no identifiable cause, associated with clinical manifestations [1].

The Working Conference on Eosinophil Disorders and Syndromes updates the criteria and definitions for eosinophilic disorders. They defined HE as >1.5 eosinophils × 109/L blood on two examinations (interval ≥ one month) and/or tissue HE. HES includes (i) criteria for peripheral blood (HE) fulfilled, (b) organ damage and/or dysfunction attributable to tissue HE, and (c) exclusion of other disorders or conditions as the major reason for organ damage [1]. HES is rare with an estimated incidence of 0.036 per 100,000. The usual age of diagnosis for HES is 20-50 years old [4].

Before retaining the diagnosis of HES, a significant number of diagnoses should be eliminated. From a dermatological perspective, it is necessary to look for dermatological lesions suggestive of a specific dermatological pathology with histopathological confirmation such as autoimmune bullous dermatoses, eczema, or atopic dermatitis [5]. When the cutaneous manifestations do not allow a diagnosis, the etiological investigation must be systematic. We will always keep in mind the major etiological lines: infectious diseases; drug, toxic, or allergic causes; neoplasias or malignant hemopathy; and systemic diseases [5].

Lymphocytic HES is a subtype of HES. It was first described by allergists and dermatologists examining patients with erythroderma and idiopathic eosinophilia [3]. It is a chronic clonal indolent T-cell lymphoproliferative disorder in which mature peripheral T cells secrete high amounts of interleukin 5 (IL-5), leading to the polyclonal expansion of eosinophils. The most well-known immunophenotype is CD3-CD4+, which is the case in our patient [6].

Complications of HE often involve the skin, lungs, heart, spleen, and nervous system. However, skin manifestations remain the most common [7]. Dermatologists must be vigilant in certain skin disorders. We cite, for example, pruritic erythematous papules, urticaria, angioedema, dermographism, mouth and genital ulcers, erythema annulare centrifugum, acral bullae, and erythroderma. Histopathological examination of the skin lesion is generally nonspecific, with inconstant eosinophilic infiltration [2].

Corticosteroids are the first therapeutic line in treating CD3-CD4+ L-HES [6,7]. Although response rates to glucocorticoids are generally high, 15% of patients do not respond at all [8], which is the case in our patient. Stokes et al. suggest that increased serum IL-5 may lead to glucocorticoid resistance in some patients with HES by impairing glucocorticoid-induced eosinophil apoptosis [8]. So, If a steroid-sparing therapy is needed, because of corticosteroid dependency or resistance, interferon-α, cyclosporin A, hydroxycarbamide, imatinib, and mepolizumab are appropriate therapeutic choices to control clinical manifestations and HE and sparing steroid consumption [6,9].

## Conclusions

HES requires early diagnosis and treatment to prevent fatal complications secondary to deep organ damage. However, the diagnosis is often delayed due to pleomorphic dermatological manifestations. In all cases of erythroderma or other cutaneous manifestations associated with eosinophilia, practitioners should consider HES as a differential diagnosis.

## References

[1] Kahn JE, Groh M, Lefèvre G (2017). (A critical appraisal of) classification of hypereosinophilic disorders. Front Med (Lausanne).

[2] Merlotto MR, Cantadori LO, Sakabe D, Miot HA (2018). Case for diagnosis. Erythroderma as manifestation of hypereosinophilic syndrome. An Bras Dermatol.

[3] Dou C, Chen LY, Dutz JP (2019). Lymphocyte-variant hypereosinophilic syndrome presenting as chronic dermatitis and responding to mycophenolic acid. JAAD Case Rep.

[4] Shomali W, Gotlib J (2019). World Health Organization-defined eosinophilic disorders: 2019 update on diagnosis, risk stratification, and management. Am J Hematol.

[5] Aubert H, Hamidou M, Barbarot S, Piriou N, Lefebvre M, Néel A (2020). Cutaneous hypereosinophilia… Hypereosinophilic syndromes. (Article in French). Ann Dermatol Venereol.

[6] Lefèvre G, Copin MC, Staumont-Sallé D (2014). The lymphoid variant of hypereosinophilic syndrome: study of 21 patients with CD3-CD4+ aberrant T-cell phenotype. Medicine (Baltimore).

[7] Mahajan VK, Singh R, Mehta KS, Chauhan PS, Sharma S, Gupta M, Rawat R (2014). Idiopathic hypereosinophilic syndrome: a rare cause of erythroderma. J Dermatol Case Rep.

[8] Stokes K, Yoon P, Makiya M (2019). Mechanisms of glucocorticoid resistance in hypereosinophilic syndromes. Clin Exp Allergy.

[9] Choi C, Moller D, Tan J (2020). Pegylated interferon alpha 2a is an effective and well-tolerated treatment option for lymphocyte-variant hypereosinophilic syndrome. Br J Haematol.

